# Microanatomy of the female reproductive system of the viviparous freshwater whipray *Fluvitrygon signifer* (Chondrichthyes: Myliobatiformes: Dasyatidae). II. The genital duct

**DOI:** 10.1186/s40850-021-00070-0

**Published:** 2021-05-05

**Authors:** Nittaya Somsap, Nopparat Srakaew, Kannika Chatchavalvanich

**Affiliations:** Department of Zoology, Faculty of Science, Kasetsart University, Bangkok, 10900 Thailand

**Keywords:** White-edge freshwater whipray, Chondrichthyes, Microanatomy, Histochemistry, Genital duct, Reproduction

## Abstract

**Background:**

Fundamental knowledge on microscopic structures of the whole female chondrichthyan genital ducts from a single species remains unavailable. The present study describes microanatomy of the entire female genital duct (anterior oviduct, oviducal gland, uterus and vagina) of the freshwater dasyatid *Fluvitrygon signifer*.

**Results:**

The females have only the left genital duct. The genital ducts reveal histological variation among individuals in terms of tissue organization, histochemical profiles and secretory activities. The anterior oviducts of mature females possess branched mucosal folds and exhibit dynamic relationship between production and secretion of secretory substances, while those of immature/regenerating females have short, unbranched mucosal folds and inactive secretory activities. The oviducal gland comprises glandular tubules, which show histological and histochemical heterogeneity and, thus, can be classified into three types. The uterus is categorized into five patterns principally based on histological features of the trophonematal and uterine mucosa. The vagina displays different histochemical reactions, likely reflecting various degrees of glycosylation of secretory granules.

**Conclusions:**

The genital ducts of the females of *F. signifer* show differential microscopic and histochemical characteristics, indicating their different reproductive statuses.

## Background

Like other vertebrates, the female reproductive system of the Chondrichthyes generally comprises the ovaries and the genital ducts. The latter are further subdivided into distinct functional regions, namely the ostium, anterior oviduct, oviducal gland, isthmus and uterus [1–3]. In some species, the posterior region of the paired uteri fuse to become a common vagina and the posterior part of the uterus transforms into a constricted, muscularized sphincter, called the uterine cervix [1, 4, 5]. However, terminology for the reproductive segment posterior to the uterus has been adopted inconsistently and interchangeably by serveral investigators, as the cloaca [2–4, 6–13], the urogenital sinus [1, 12, 14–16] and the vagina [17–24]. Herein, this region is referred to as the vagina. In general, the chondrichthyan female genital ducts play multiple roles in collecting, transporting and storing gametes, forming the egg coverings, supplying nutrients and oxygen to embryos, removing waste products from these latter ones in case of viviparous species, providing protective milieu for fertilization and embryonic development, serving as a passageway for delivering offspring to the exterior and accommodating the male intromittent organ during copulation [4, 25].

The white-edge freshwater whipray *Fluvitrygon signifer* (Myliobatiformes: Dasyatidae) is a reclassified taxon of *Himantura signifer* Compagno and Roberts, 1982 based on a recent study [26]. It is rare and threatened according to IUCN [27]. This dasyatid stingray mainly inhabits the freshwater ecosystems of the Indo-Malay Archipelago, with their first record in the Kapuas (Kalimantan, Indonesia) and subsequently in Indragiri river (Riau, Sumatra, Indonesia), the Perak river (western Peninsular Malaysia), Chao Phraya river (Thailand) [28], and Musi river basin and Musi river drainage, South Sumatra (Indonesia) [29]. The young are born with their disc width of 11–12 cm, while males and females reach sexual maturity at their disc widths of ~ 21–23 and 25–26 cm, respectively [30, 31]. Like other myliobatiforms, *F. signifer* is matrotrophic viviparous, with the uterine embryos gaining nutrients from lipid- and protein-rich histotroph secreted from villus-like uterine appendages, called trophonemata [28, 32, 33].

Insight into the overall process of reproduction of *F. signifer* will contribute to the first fundamental step to improved knowledge on its reproductive biology that could be useful in the future to better understand the reproductive cycle, leading to developing effective management and conservation strategies for protection of this species. One essential aspect of their reproductive biology integral to reproductive functions is microanatomy of the reproductive system. The microanatomical structures of the ovary of *F. signifer* has been recently described [34], but those of the female genital ducts have not been reported. As far as is known, basic knowledge on the microscopic structures of the myliobatiform female genital ducts has been gathered from several studies of various species belonging to different families, including three marine urotrygonids [15, 35–38], one marine gymnurid [39], one marine myliobatid [40], seven freshwater potamotrygonids [41, 42], one marine rhinopterid [43] and four marine dasyatids [6, 17, 35, 44, 45]. However, detailed histological and histochemical descriptions of the entire genital ducts and information on body morphometrics related to maturity statuses from a single species are still not available. This study aimed to investigate the microanatomy of the genital ducts of the freshwater dasyatid, *Fluvitrygon signifer*.

## Results

### General structures

Body morphometrics and maturity scale of *Fluvitrygon signifer* are shown in Table 1. The females have only the left genital duct that is divided into four regions from the anterior to the posterior direction: the anterior oviduct, the oviducal gland, the uterus and the vagina. The genital duct is constituted by three concentric tissue layers from the inside to the outside: mucosa, muscularis and adventitia.

**Table 1:** Body morphometrics and ovarian microscopic descriptions of *Fluvitrygon signifer*

Animals	Date of collection	Body weight (g)	Ovary weight (g)	Disc width (cm)	Disc length (cm)	Previtellogenic follicle	Vitellogenic follicle	Postovulatory follicle	Maturity scale*
F1	02/25/99	864	1.760	31.0	28.0	Present	Absent	Absent	Regenerating
F2	04/26/99	870	2.080	30.0	28.2	Present	Present	Present	Mature
F3	06/29/99	722	2.282	28.0	26.0	Present	Present	Present	Mature
F4	09/22/99	841	2.493	31.0	29.0	Present	Present	Present	Mature
F5	11/30/99	1270	6.383	35.0	33.5	Present	Present	Present	Mature
F6	02/03/00	220	0.643	20.0	17.5	Present	Absent	Absent	Immature
F7	02/14/00	210	0.649	19.5	18.0	Present	Absent	Absent	Immature
F8	10/18/00	665	5.534	28.0	26.5	Present	Present	Present	Mature

*Assessment for maturity scale is based on disc width [30, 31] and ovarian microscopic examination [34, 46]

### Microscopic structures of the genital ducts

#### Anterior oviduct

**Fig. 1:**
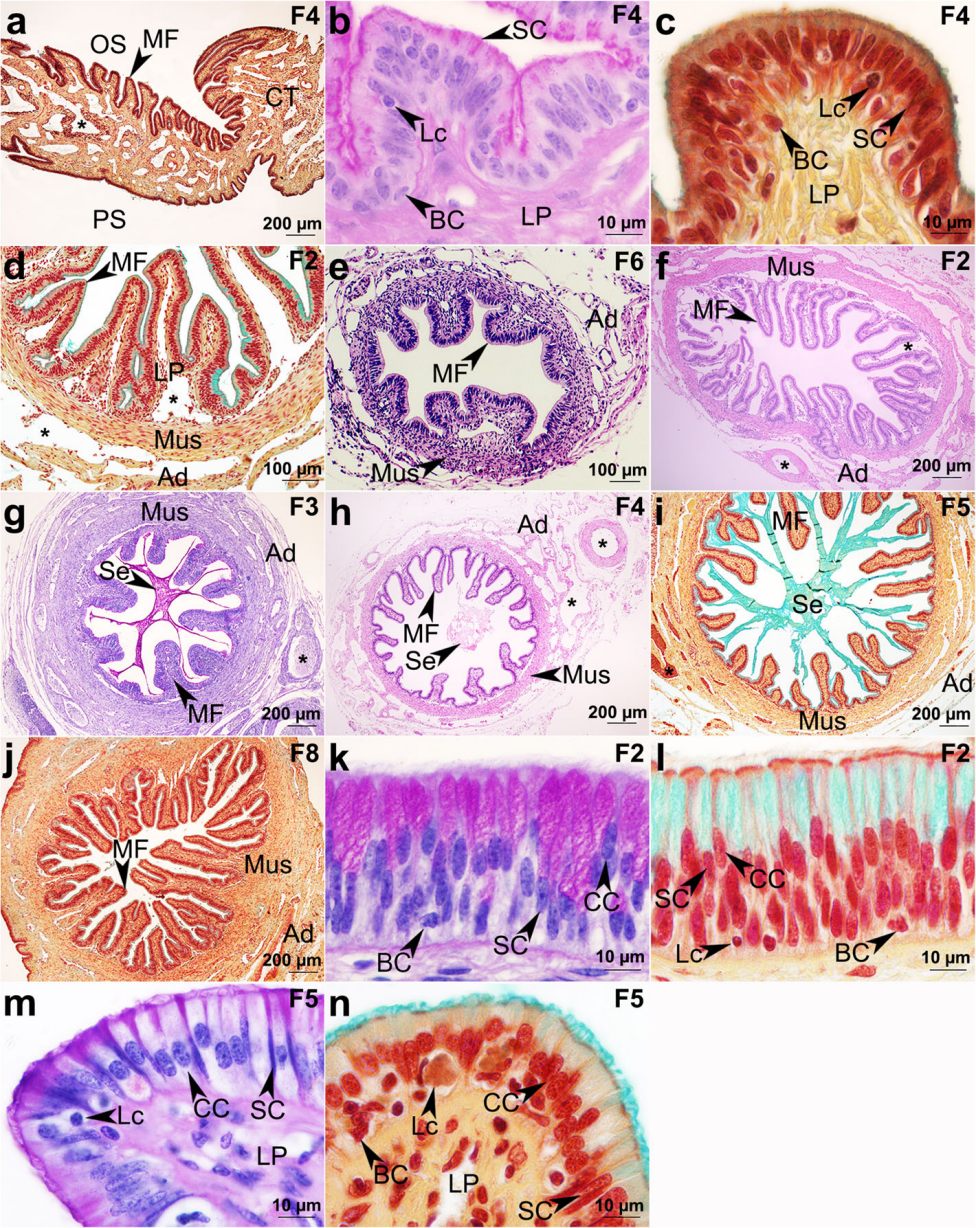
Microscopic structures of the anterior oviducts of *Fluvitrygon signifer*. **a** Fimbriated ostium bordered by an ovarian surface (OS) and a peritoneal surface (PS). **b, c **Mucosa of the fimbria showing epithelial cells. **d** Oviductal wall comprising three tissue layers. **e**-**j **Anterior oviducts of F6 **(e**), F2 (**f**), F3 (**g**), F4 (**h**), F5 (**i**) and F8 (**j**) females. **k**-**n** Oviductal mucosa of F2 (**k**,** l**) and F5 (**m**, **n**) specimens demonstrating distribution of glycoconjugate materials. Specimen codes are labeled in their corresponding photomicrographs. Abbreviations: Ad, adventitia; BC, basal cell; CC, ciliated cell; CT, connective tissue; Lc, leukocyte; LP, lamina propria; MF, mucosal fold; Mus, muscularis; SC, secretory cell; Se, secretory material; *, blood vessel. Stains: **a**, **c**, **d**, **i**, **j**, **l**, **n** = modified Movat’s pentachrome; **b**, **g**, **k**,** m** = PAS-H; **e**, **f**, **h **= H&E

The anterior portion of the oviduct is a fringed funnel surrounding an ostium. This fimbriated tissue has the ovarian and peritoneal surfaces, with the former facing the ovary and having long mucosal folds, while the latter opposing the peritoneal cavity and possessing short mucosal folds (Fig. 1a). The fimbria is lined by a pseudostratified columnar epithelium, consisting of three cell types: secretory columnar cells, basal cells and leukocytes (Fig. 1b, c). The secretory cell cytoplasm has affinity for PAS and to a lesser extent AB pH 2.5 in pentachrome staining, suggesting accumulation of neutral and carboxylated acid glycoconjugates, respectively (Fig. 1b, c). The core of the fimbria comprises mainly dense irregular connective tissues that are extensively vascularized (Fig. 1a).

The anterior oviduct contains branched mucosal folds (Fig. 1d-j). The mucosa is composed of a pseudostratified ciliated columnar epithelium and a lamina propria (Fig. 1d, m, n). Four epithelial cell types are identifiable: ciliated cells, columnar secretory cells, basal cells and leukocytes (Fig. 1k-n). Ciliated cells bear central, ovoid nuclei and apical cilia (Fig. 1k-n). Columnar secretory cells have a pyriform-like shape, basally-located ovoid nuclei, and neutral and carboxylated acid mucopolysaccharides in the cytoplasm, as demonstrated by PAS and AB pH 2.5 in pentachrome staining, respectively (Fig. 1k-n), but not sulfated acid mucopolysaccharides based on AB pH 1.0 histochemistry. PAS reactivity of the oviduct tissues is not inhibited upon pretreatment of the tissue sections with diastase, as compared to those without enzymatic treatment (data not shown). Basal cells have irregular nuclei and are located at the basal region of the epithelium, while leukocytes are surrounded by pericellular spaces and scattered among other epithelial cells (Fig. 1k-n). The lamina propria is made of loose collagenous connective tissues (Fig. 1d, m, n). The muscularis is a fibromuscular layer, containing circularly-oriented smooth muscle cells distributed among dense collagenous tissues (Fig. 1d). The adventitia is a vascularized loose connective tissue (Fig. 1d-j).

The anterior oviduct reveals histological variation among specimens. The oviducts of F1, F6 and F7 females have thin wall, short mucosal folds and inactive secretory activities (Fig. 1e). In F2 specimen, the lamina propria and the adventitia are highly vascularized (Fig. 1d, f). The oviducts of F3, F4 and F5 females carry luminal secretion (Fig. 1g-i), which reacts to PAS (Fig. 1g) and AB pH 2.5 in pentachrome staining (Fig. 1i), confirming a mixture of neutral and carboxylated acid glycoproteins/mucopolysaccharides. The mucosal folds of F3 female are long and stout (Fig. 1g) whereas those of F4 and F5 females are branched and slender (Fig. 1h, i). The folds are numerous and highly branched in F8 female (Fig. 1j). In F2 and F8 females, glycoconjugate materials tend to be accumulated in the secretory cell cytoplasm (Fig. 1k, l) with little luminal secretion (Fig. 1d, f, j), while those of F3, F4 and F5 specimens are mainly secreted into the lumen (Fig. 1g-i) with less storage in the secretory cell cytoplasm (Fig. 1m, n), implying dynamic statuses between accumulation and secretion of secretory materials. Sulfated glycoconjugate materials are not detectable in these cells upon AB pH 1.0 staining.

#### Oviducal gland

**Fig. 2:**
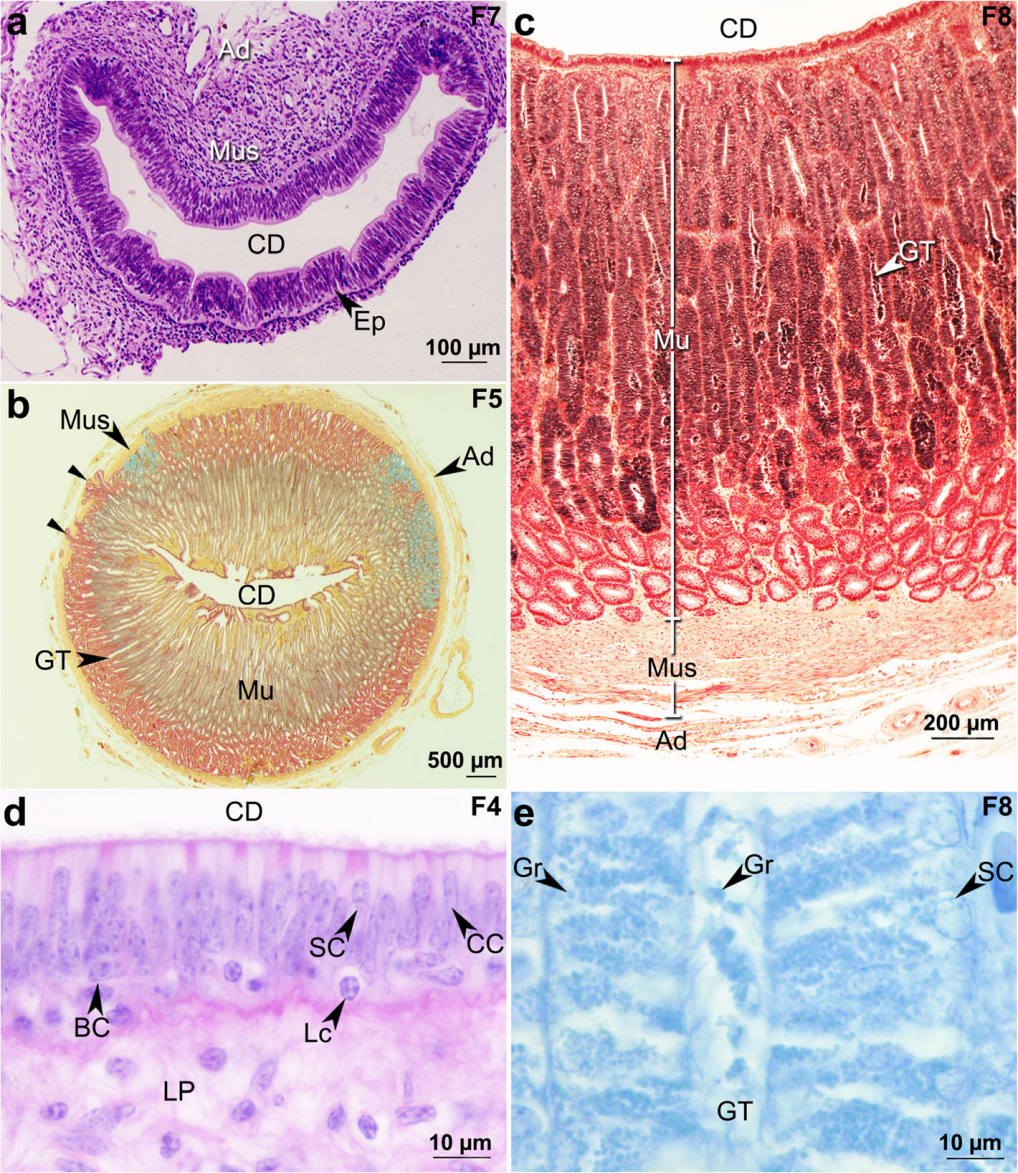
Histological structures of the oviducal glands of *Fluvitrygon signifer*. **a** Oviducal gland without glandular tubules of F7 female. **b** Oviducal gland of mature females with multiple glandular tubules (GT) organized in a centripetal manner around a central duct (CD); note extension of the glandular tubules into the muscularis (Mus), as indicated by black triangles. **c** Oviducal gland wall with numerous glandular tubules occupying almost the entire thickness of the mucosa. **d** Mucosa of the central duct composed of epithelial cells overlying a lamina propria (LP). **e **A glandular tubule showing protein compositions of secretory granules (Gr) in secretory cells (SC) and in the tubular lumen. Photomicrographs are indicated with corresponding specimen codes. Abbreviations: Ad, adventitia; BC, basal cell; CC, ciliated cell; Ep, epithelium; Lc, leukocyte; Mu, mucosa. Stains: **a** = H&E; **b**, **c** = modified Movat’s pentachrome; **d** = PAS-H; **e** = bromophenol blue

**Fig. 3:**
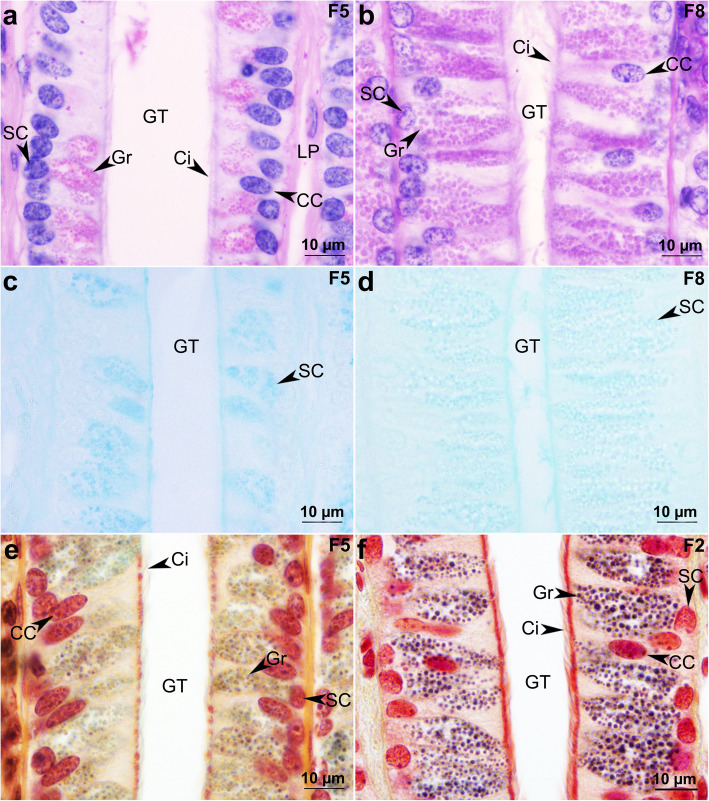
Histological and histochemical characteristics of the upper quarter region of glandular tubules. The upper quarter region of type I and II (**a**, **c**, **e**) and type III (**b**, **d**, **f**) glandular tubules. Specimen codes are provided in their corresponding photomicrographs. Abbreviations: CC, ciliated cell; Ci, cilia; Gr, granule; GT, glandular tubule; SC, secretory cell. Stains: **a**, **b** = PAS-H; **c**, **d** = AB pH 2.5; **e**, **f**= modified Movat’s pentachrome

The oviducal glands of immature females are a straight tube without glandular tubules (Fig. 2a). In mature females, the mucosa is the thickest of the oviducal gland proper (Fig. 2b, c). This layer houses multiple simple straight/branched glandular tubules as structural secretory units that span the entire thickness of the mucosa and are organized in a centripetal manner with respect to a central duct (Fig. 2b). Some glandular tubules extend into the muscularis (Fig. 2b). The central duct is lined by a pseudostratified columnar epithelium, composed of four cell types: secretory cells, ciliated cells, basal cells and leukocytes (Fig. 2d). Secretory cells have ovoid nuclei and their supranuclear cytoplasm is reactive to PAS (Fig. 2d) and AB pH 2.5 in pentachrome histochemistry (data not shown). Underneath the ductal and tubular epithelia is the lamina propria, constituted by loose connective tissues (Figs. 2d, 3a). The muscularis contains smooth muscle fibers lying in a circular disposition and interspersed with collagenous connective tissues (Fig. 2c). The adventitia is a vascularized loose connective tissue (Fig. 2a-c).

**Fig. 4:**
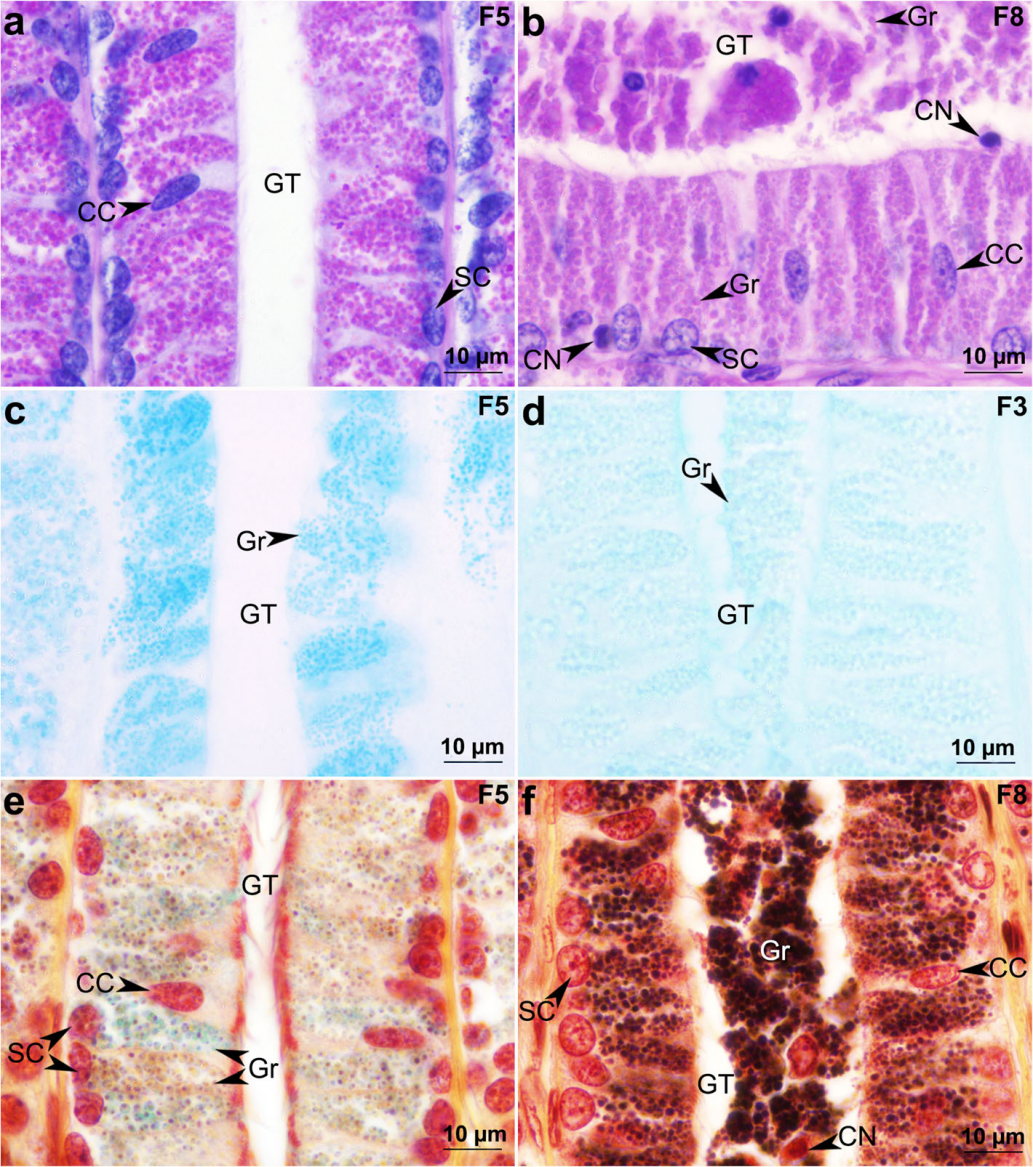
Histochemistry of the middle half region of glandular tubules.The middle half region of type I and II (**a**, **c**, **e**) and type III (**b**, **d**, **f**) glandular tubules. Photomicrographs are indicated with corresponding specimen codes. Abbreviations: CC, ciliated cell; CN, condensed nucleus; Gr, granule; GT, glandular tubule; SC, secretory cell. Stains: **a**, **b** = PAS-H; **c**, **d** = AB pH 2.5; **e**, **f** = modified Movat’s pentachrome

**Fig. 5:**
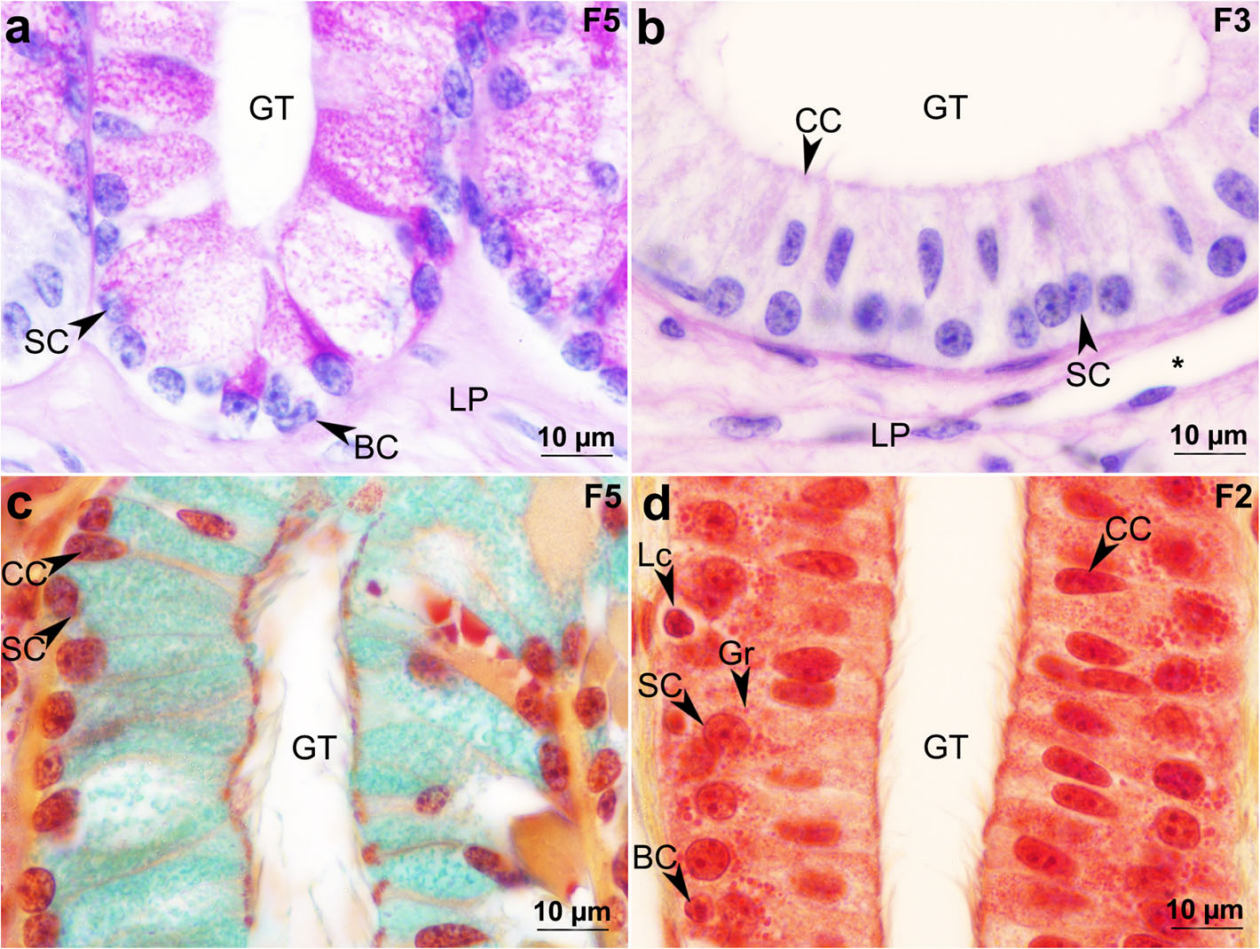
Histological and histochemical profiles of the lower quarter region of glandular tubules. The lower quarter region of type I (**a**, **c**) and type II and III (**b**, **d**) glandular tubules. Photomicrographs are labeled with corresponding specimen codes. Abbreviations: BC, basal cell; CC, ciliated cell; Gr, granule; GT, glandular tubule; Lc, leukocyte; LP, lamina propria; SC, secretory cell; *, blood vessel. Stains: **a**, **b** = PAS–H; **c**, **d** = modified Movat’s pentachrome

The glandular tubules are lined by a pseudostratified columnar epithelium comprising secretory columnar cells, ciliated cells, basal cells and leukocytes (Fig. 2e, 3, 4, and 5). Secretory cells possess basal, ovoid-to-spherical nuclei and supranuclear granules (Figs. 2e, 3, 4, and 5) that contain proteins (Fig. 2e), neutral glycan moieties (Figs. 3a, b, 4a, b, 5a, b) and carboxylated acid glycan moieties (Figs. 3c, d, 4c, d, 5c). Ciliated cells have oval nuclei located at higher levels than those of the secretory cells (Figs. 3a, b, e, f, 4a, b, e, f, 5). Basal cells with irregular nuclei are detectable at the epithelial basal compartment (Fig. 5a, d). Leukocytes characterized by pericellular spaces are scattered among other epithelial cell types (Fig. 5d). The oviducal glands display histological and histochemical heterogeneity within and among individual tubules (Figs. 2b, 3, 4, and 5), possibly reflecting different secretory statuses of the tubules.

Herein, each glandular tubule is divided into three approximate regions along its entire length from the adluminal to the basal aspects, viz. upper quarter, middle half and lower quarter regions. Notably, there exist three histological and histochemical patterns of the glandular tubules (types I-III) in the specimens (Figs. 3, 4, and 5). The upper quarter region of type I and II glandular tubules has short secretory cells with secretory granules (Fig. 3a, c, e) whereas the middle half region of the tubules carries numerous granules occupying the secretory cell cytoplasm, with fewer granules secreted into the lumen (Fig. 4a, c, e). In contrast, secretory cells of the upper region of type III glandular tubules are taller and store more cytoplasmic granules than those of type I and II tubules (Figs. 3b, d, f versus a, c, e). In the middle half region of type III glandular tubules, a number of granules engorge the secretory cell cytoplasm, causing cell expansion and difficulty in defining cell boundary, and are released into the lumen, indicating active tubular secretion (Fig. 4b, d, f). Some secretory cells nuclei are condensed and shed into the tubular lumen (Fig. 4b, f). Further, it is noted that staining patterns of the granules in the upper quarter and middle half regions from consecutive histological sections are divided into two groups, following AB pH 2.5 and pentachrome histochemistry. In the first group, as seen in type I and II glandular tubules, the granules show moderate to strong reactions with AB (Figs. 3c, 4c) and reveal different colorations upon pentachrome staining, i.e. blue, reddish brown and brown colors (Figs. 3e, 4e). In the second group, as observable in type III glandular tubules, the granules are faintly stained with AB pH 2.5 (Figs. 3d, 4d) and become black after pentachrome staining (Figs. 3f, 4f). In the lower quarter region of the glandular tubules, the granules in the secretory cells of type I glandular tubules show intense magenta staining with PAS (Fig. 5a) and are strongly alcianophilic upon both a monochrome AB pH 2.5 (data not shown) and AB pH 2.5 in pentachrome staining (Fig. 5c). In contrast, the granules in the secretory cells at the lower quarter region of type II and III tubules are moderately reactive to PAS (Fig. 5b) and stained red, following pentachrome histochemistry (Fig. 5d). The histological and histochemical patterns of the glandular tubules vary among specimens, with type I and II glandular tubules present only in F5 female and type III tubules found in F2, F3, F4, F5 and F8 females. Pretreatment of the oviducal gland sections with diastase does not affect to affinity of the granules for PAS (data not shown). Moreover, the secretory granules in all specimens are not reactive to AB pH 1.0.

#### Uterus

**Fig. 6:**
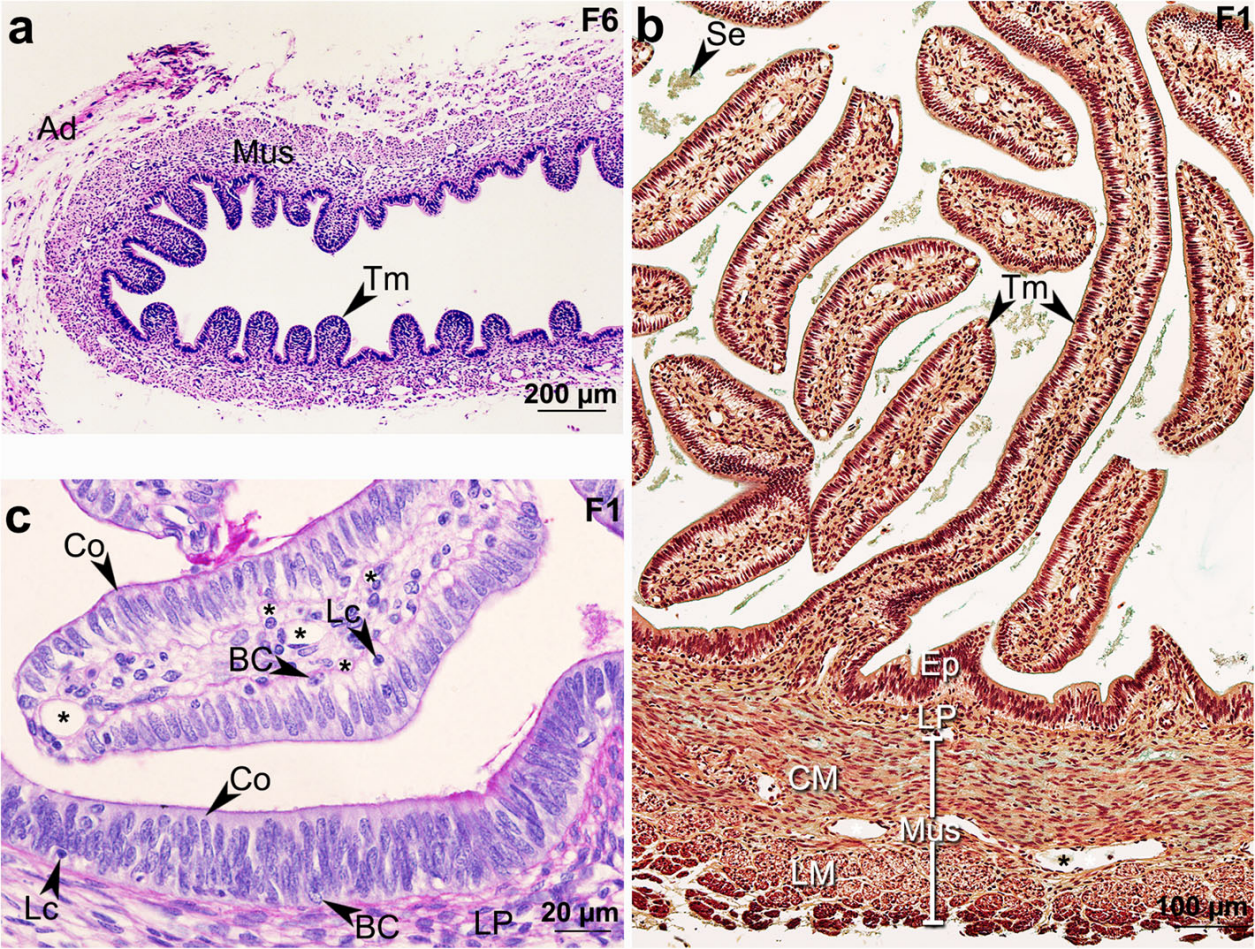
Uterine microanatomy of immature and regenerating females of *Fluvitrygon signifer*. **a** Uterus of F6 female with small and short trophonemata (Tm). **b**, **c** Uterus of F1 female showing the uterine wall (**b**), and uterine and trophonematal epithelia (**c**). Micrographs are designated with their corresponding specimen codes. Abbreviations: Ad, adventitia; BC, basal cell; CM, circular smooth muscle; Co, columnar cell; Ep, epithelium; Lc, leukocyte; LM, longitudinal smooth muscle; LP, lamina propria; Mus, muscularis; Se, secretory material; *, blood vessel. Stains: **a** = H&E; **b** = modified Movat’s pentachrome; **c** = PAS–H

**Fig. 7:**
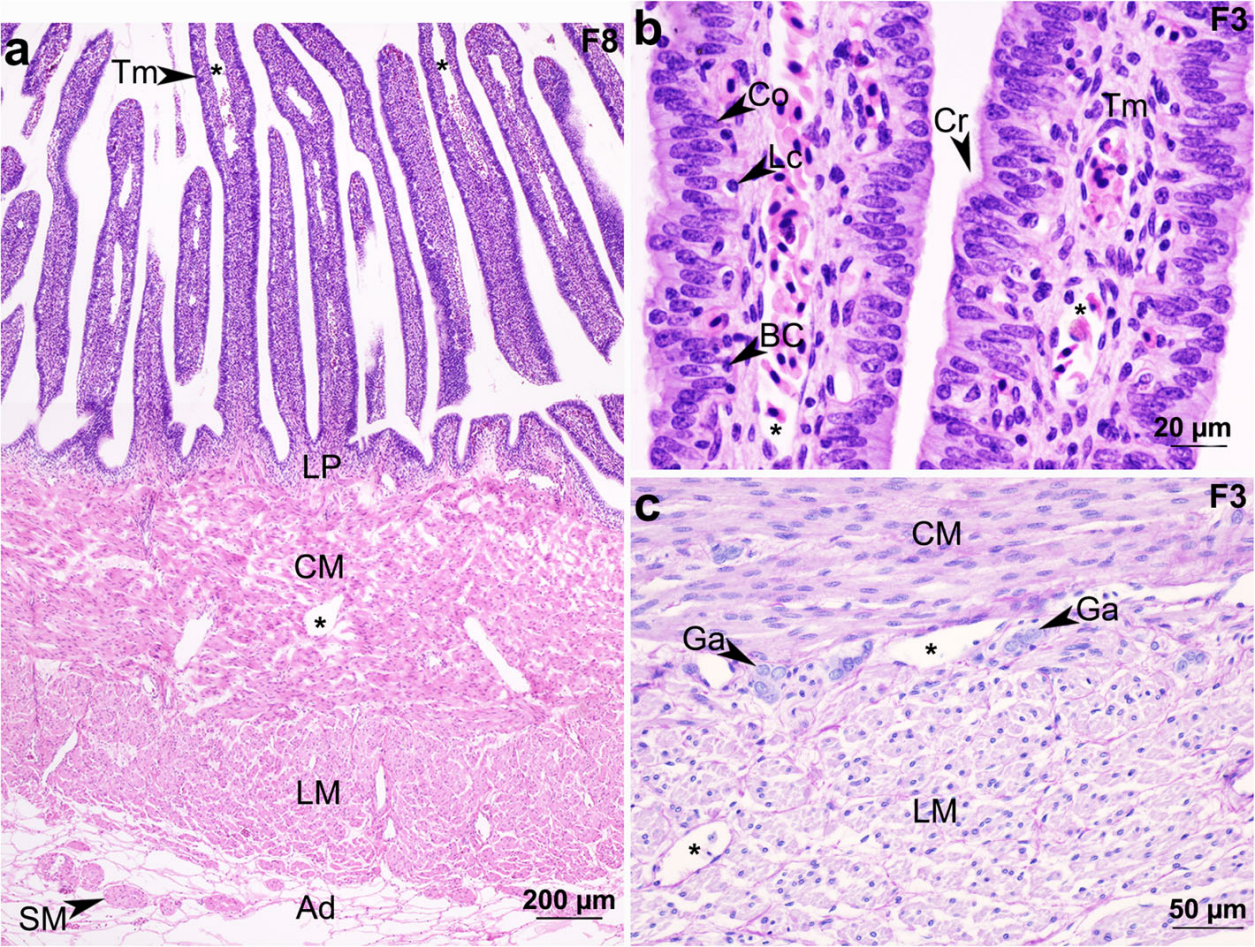
Uterine microscopic structures of F3 and F8 females of *Fluvitrygon signifer*. **a** Uterine wall of F8 female. **b**, **c** Uterus of F3 female revealing trophonemata (Tm) with glandular crypts (Cr) (**b**) and uterine muscularis (**c**). Photomicrographs are specified with corresponding specimen codes. Abbreviations: Ad, adventitia; BC, basal cell; CM, circular smooth muscle; Co, columnar cell; Ga; ganglion cell; Lc, leukocyte; LM, longitudinal smooth muscle; LP, lamina propria; SM, smooth muscle; *, blood vessel. Stains: **a**, **b** = H&E; **c** = PAS–H

**Fig. 8:**
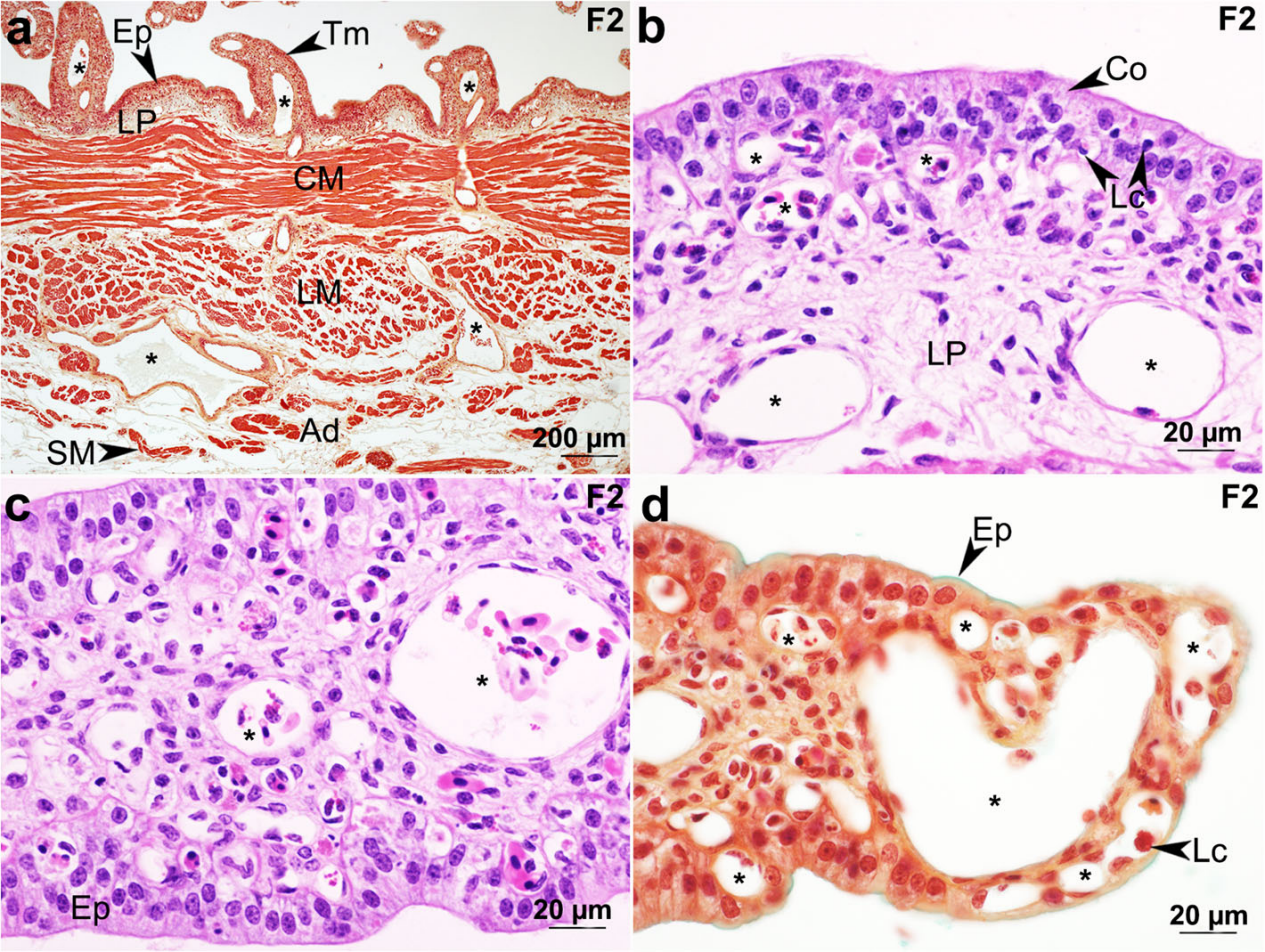
Uterine histology of F2 female of *Fluvitrygon signifer*. The entire uterine wall (**a**), uterine mucosa (**b**) and trophonemata (Tm) (**c**, **d**). Specimen codes are used to indicate their corresponding photomicrographs. Abbreviations: Ad, adventitia; CM, circular smooth muscle; Co, columnar cell; Ep, epithelium; Lc, leukocyte; LM, longitudinal smooth muscle; LP, lamina propria; SM, smooth muscle; *, blood vessel. Stains: **a**, **d** = modified Movat’s pentachrome; **b**, **c** = H&E

**Fig. 9:**
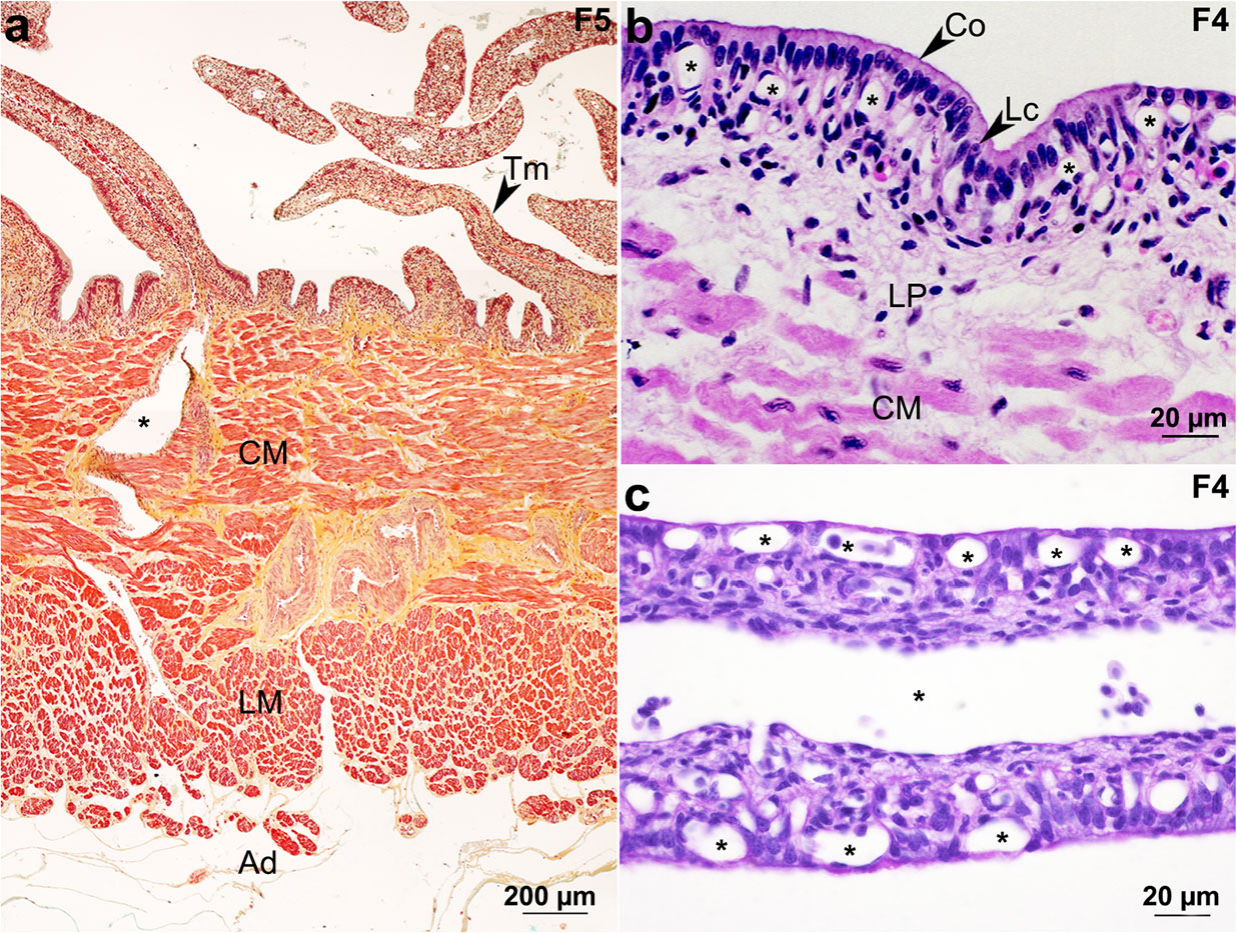
Uterine microscopic structures of F4 and F5 females of *Fluvitrygon signifer*. Uterus of F4 and F5 females having the thickest uterine wall (**a**), uterine epithelium atop numerous blood vessels (**b**) and trophonemata (Tm) with a thin epithelium overlying blood vessels (**c**). Micrographs are designated with their corresponding specimen codes. Abbreviations: Ad, adventitia; CM, circular smooth muscle; Co, columnar cell; Lc, leukocyte; LM, longitudinal smooth muscle; LP, lamina propria; *, blood vessel. Stains: **a** = modified Movat’s pentachrome; **b** = H&E; **c** = PAS–H

The uterus is a muscular tube (Figs. 6, 7, 8, and 9). The mucosa comprises a pseudostratified columnar epithelium and a lamina propria (Figs. 6c, 8b, 9b). The uterine epithelium consists of three cell types, i.e., columnar cells, basal cells and leukocytes (Figs. 6c, 8b, 9b). The mucosa also extends into the uterine lumen and becomes trophonemata (Figs. 6a, b, 7a, 8a, 9a). A central vessel in the trophonematal core ramifies into vascular plexuses and courses along the trophonematal margin, thus giving off peripheral arterioles and capillaries underneath the trophonematal epithelium (Figs. 8c, d, 9c). The muscularis comprises two orthogonal muscular sheets disposed into the inner circular and outer longitudinal muscular tunics (Figs. 6b, 7a, c, 8a, 9a). Autonomic ganglia are intercalated between the two musculatures (Fig. 7c). The adventitia is a vascularized loose connective tissue (Figs. 6a, 7a, 8a, 9a). Scattered bundles of circular and longitudinal muscles are also observable in the adventitia (Figs. 7a, 8a).

**Fig. 10:**
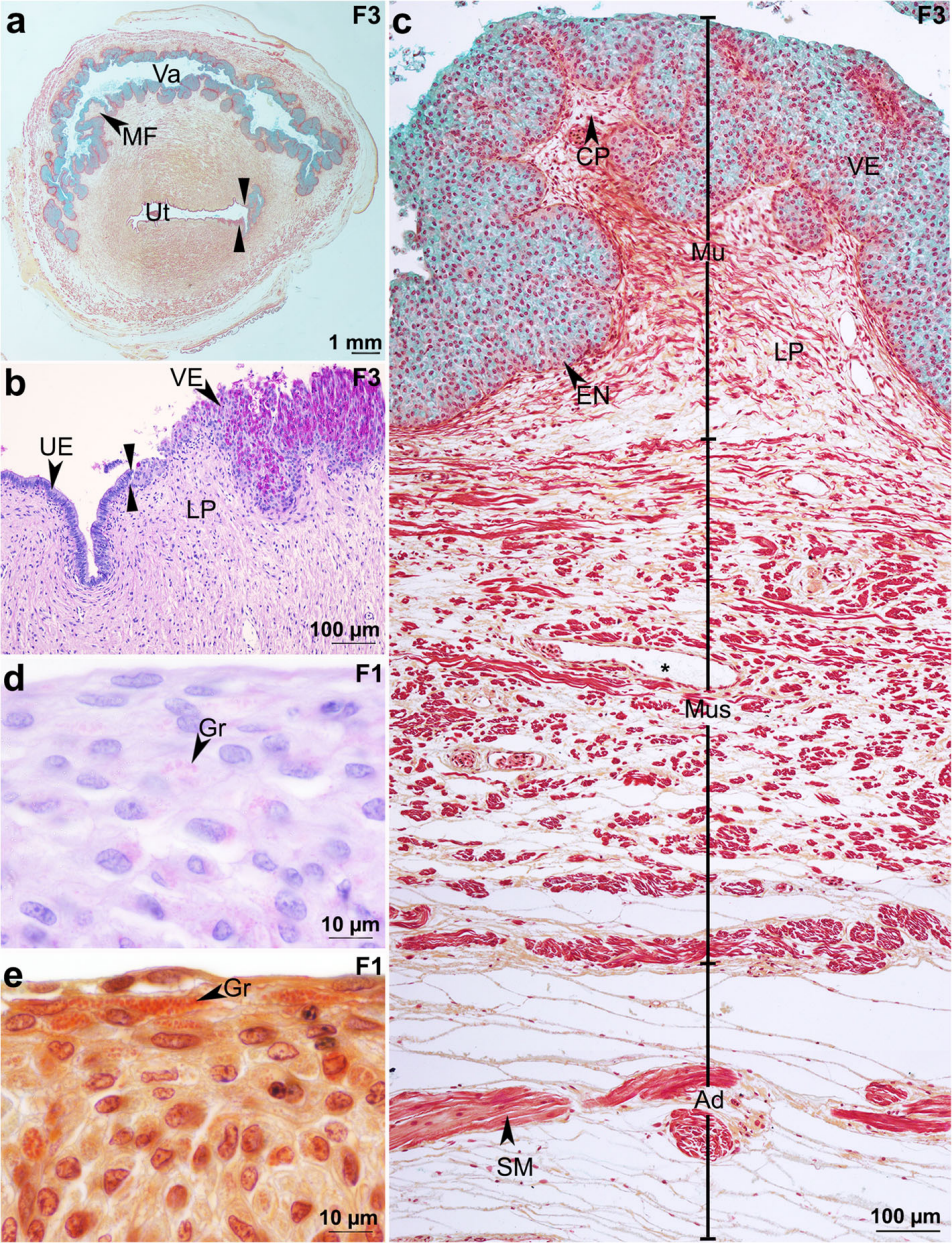
Microanatomical structures of the vagina of *Fluvitrygon signifer*. **a**, **b** Cross sectional view of the anterior region of the vagina (Va) and the posterior region of the uterus (Ut). **b** Abrupt histological transition of the mucosa from the uterine pseudostratified columnar epithelium (UE) to the vaginal stratified squamous/transitional-like epithelium (VE), as indicated by two opposing black triangles. **c** Vaginal wall with a mucosal fold and epithelium notch (EN). **d**, **e** Vaginal epithelia of F1. Specimen codes are used to specify their corresponding photomicrographs. Abbreviations: Ad, adventitia; CP: connective tissue papilla; Gr, granule; LP, lamina propria; MF, mucosal fold; Mu, mucosa; Mus, muscularis; SM, smooth muscle; *, blood vessel. Stains: **a**, **c**, **e** = modified Movat’s pentachrome; **b**, **d** = PAS–H

The uterus exhibits histological variation among collected specimens with five histological patterns. The first pattern shows a thin uterine wall with short trophonemata, as seen in immature F6 and F7 females (Fig. 6a). In the second pattern, the trophonemata are longer than those of the immature females and have a smooth surface of the epithelium overlying subepithelial arterioles, as found in F1 female (Fig. 6b, c). The trophonematal and uterine columnar epithelial cells have long drop-shaped nuclei and are coated with glycocalyces containing neutral glycans (Fig. 6c) and carboxylated acid glycans (data not shown). The third histological pattern of the uterus is found in F3 and F8 females (Fig. 7). The muscularis is much thicker than that of the earlier two patterns (Figs. 7a versus 6a, b). The trophonemata have a corrugated contour due to reorganization of the epithelial cells into shallow crypts (Fig. 7b). In the fourth pattern, as observed in F2 female, the trophonematal and uterine epithelia consist of low columnar cells with spherical-to-ovoid nuclei (Fig. 8b-d). The trophonemata is bordered by heterogeneous types of epithelia, i.e., pseudostratified columnar, simple cuboidal and simple squamous epithelia (Fig. 8c, d). Notably, numerous blood vessels are immediately subjacent to both epithelia (Fig. 8b, d). The trophonemata are thicker than those of the earlier patterns due to increased vascularization and cellularity in the trophonematal core (Figs. 8a, c, d versus 6, 7a). The uterine muscularis and adventitia have increased amount of loose connective tissues with dilated blood vessels and interstitial spaces (Fig. 8a). In the fifth pattern, the uterine mucosa has similar histological structures to those of F2 female (Fig. 9a, b), but the trophonematal epithelium overlying peripheral arterioles and capillaries becomes flattened, as seen in F4 and F5 specimens (Fig. 9c). The posterior region of the uterus is devoid of trophonemata (Fig. 10a).

#### Vagina

**Fig. 11:**
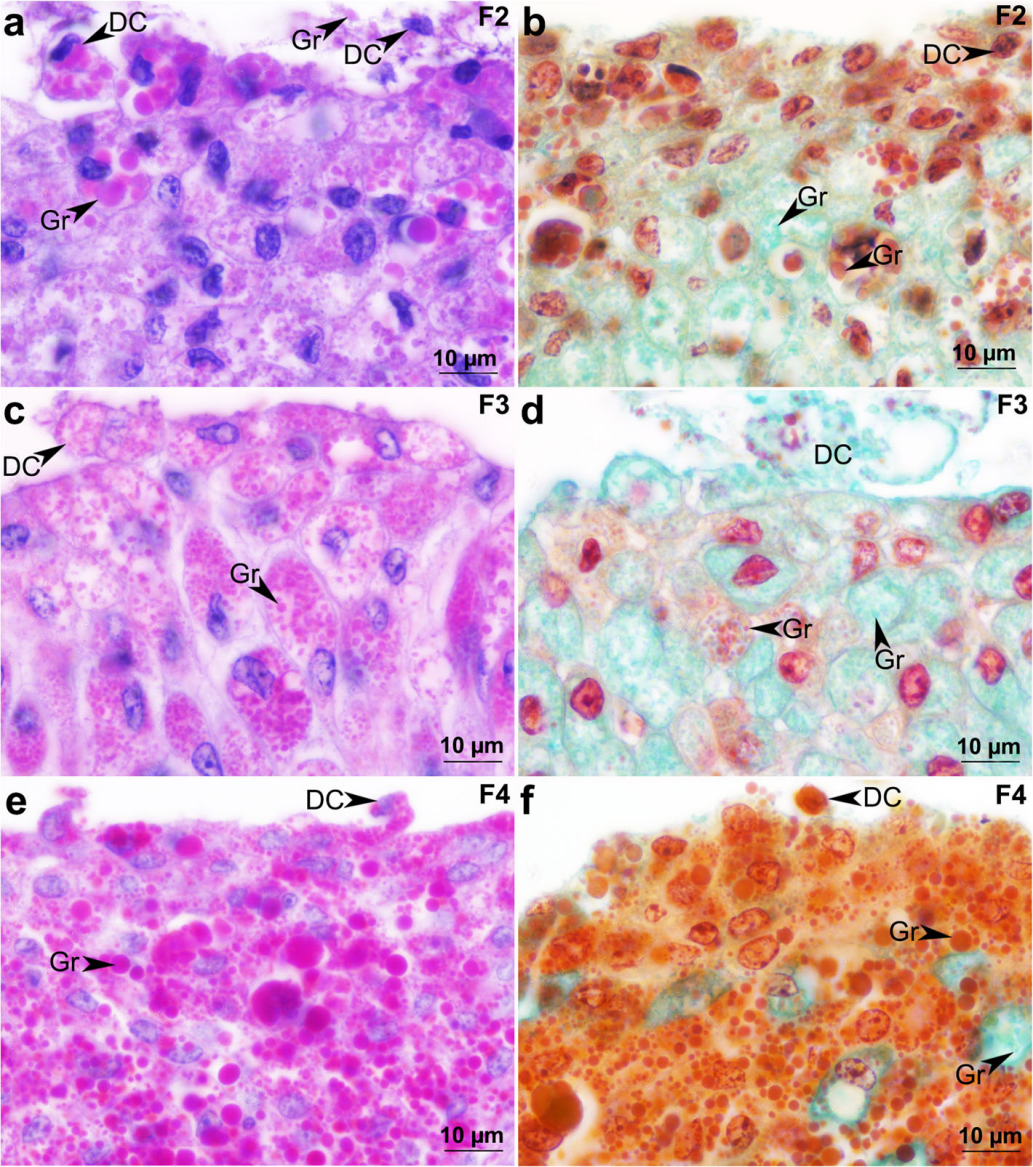
Histochemical characteristics of the vaginal epithelium of F2, F3 and F4 females of *Fluvitrygon signifer*. The vaginal epithelium of F2 (**a**, **b**), F3 (**c**, **d**) and F4 (**e**, **f**) females showing differential histochemical profiles. Photomicrographs are designated with their corresponding specimen codes. Abbreviations: DC: desquamated cell; Gr, granule. Stains: **a**, **c**, **e** = PAS–H; **b**, **d**, **f** = modified Movat’s pentachrome

**Fig. 12:**
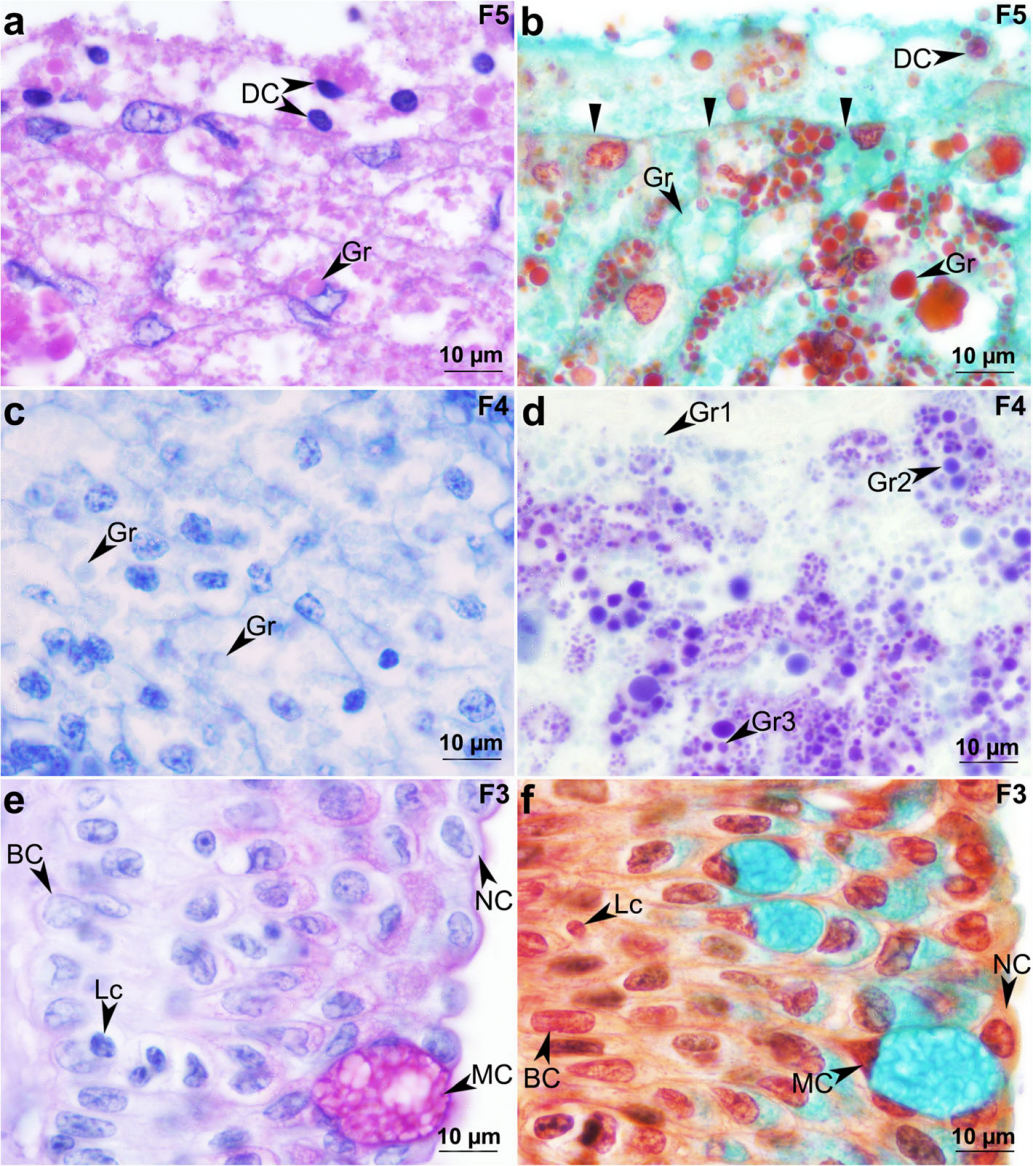
Histochemistry of the vaginal epithelium of F3, F4 and F5 females of *Fluvitrygon signifer*. The vaginal epithelium of F5 female (**a**, **b**) showing a desquamation border of the epithelium indicated by black triangles (**b**). Determination of proteins (**c**) and metachromatic property (**d**) of the granules. **e**, **f** Posterior region of the vagina having epithelial cell types. Specimen codes indicate their corresponding micrographs. Abbreviations: BC, basal cell; DC: desquamated cell; Gr, granule; Gr1–3, type 1–3 granules with different levels of metachromasia; Lc, leukocyte; MC, mucous cell; NC, non-secretory cell. Stains: **a**, **e** = PAS-H; **b**, **f** = modified Movat’s pentachrome; **c** = bromophenol blue; **d** = toluidine blue

The vagina is a fibromuscular structure (Fig. 10a, c). The uterovaginal junction reveals abrupt histological mucosal transition from the uterine pseudostratified columnar epithelium to the vaginal stratified epithelium (Fig. 10b). The epithelia at the anterior region of the vagina are of two types: a stratified squamous epithelium (Fig. 10d, e), as seen in F1, F6 and F7 females, and a transitional-like epithelium (Figs. 11, 12a, b), as found in F2, F3, F4, F5 and F8 females. The lamina propria is a loose connective tissue mixed with smooth muscle bundles (Fig. 10c). The posterior region of the vagina is lined by a transitional-like epithelium (Fig. 12e, f). Shedding of superficial epithelial cells is common among specimens (Figs. 11, 12a, b), with some desquamated cell nuclei becoming condensed (Fig. 12a, b). The epithelium invaginates into the lamina propria to become epithelial nodules flanked by connective tissue papillae (Fig. 10c). Mucosal folds are also observable (Fig. 10a). The muscularis is made of scattered bundles of the circular smooth muscles interspersed with loose connective tissues and the adventitia comprises mainly loose connective tissues with few bundles of the smooth muscles (Fig. 10c).

The epithelial cells at the anterior region of the vagina are loaded with heterogeneously-sized secretory granules that strongly react to PAS (Figs. 10d, 11a, c, e, 12a), but show negative reactivity with AB pH 1.0, indicating the presence of neutral glycans, but the absence of sulfated acid glycans, respectively. Much fewer granules are found in F1, F6 and F7 females than those in the other specimens (Figs. 10d, e versus 11, 12a-d). It is noted that PAS reactivity of these granules is not suppressed following pretreatment of the vaginal tissue sections with diastase in all specimens, confirming that the granules are not composed of glycogens (data not shown). Upon pentachrome staining, the granules are divided into two populations: blue and orange-red populations (Figs. 10e, 11b, d, f, 12b). The former is attributed to positive reaction with AB pH 2.5 in pentachrome staining, indicating existence of carboxylated acid glycans, while the latter are likely due to combined reactions with crocein scarlet-acid Fuchsin solution in pentachrome staining, suggesting the presence of other types of unknown chemical compositions. Besides, proportion of quantities between the two populations varies independently among specimens (Figs. 10e, 11b, d, f, 12b). In F1, F6 and F7 females, only small orange-red granules are found in few epithelial cells (Fig. 10e). Blue granules are more than orange-red granules, as seen in F2, F3 and F5 females (Figs. 11b, d, 12b), and the reverse is true for F4 female (Fig. 11f). However, the secretory granules are weakly reactive to bromophenol blue, indicating small quantities of proteins in the granules (Fig. 12c). Moreover, these granules display three staining patterns based on toluidine blue histochemistry, i.e., pale blue, blue and magenta colors, suggesting different levels of metachromasia possibly caused by dissimilar anionic chemical constituents among the granules (Fig. 12d). Four epithelial cell types (non-secretory cells, mucous cells, basal cells and leukocytes) are detectable at the posterior region of the vagina (Fig. 12e, f). Mucous cells accumulate neutral and carboxylated acid glycans, as demonstrated by their affinity for PAS and AB pH 2.5 in pentachrome staining (Fig. 12e, f), respectively, but not for sulfated acid glycans, as determined by their negative reaction with AB pH 1.0.

## Discussion

The female genital duct of *Fluvitrygon signifer* is subdivided into four functional regions, viz., the anterior oviduct, oviducal gland, uterus and vagina, like other chondrichthyans [4, 7, 36]. Microanatomy of the chondrichthyan oviduct displays taxon-specific variation that is not correlated with taxonomic classification, with the oviductal epithelia categorized into three types: (1) a simple ciliated columnar epithelium, as in the ratfish, *Hydrolagus colliei* [20]; the guitar fish, *Rhinobatos lentiginosus* [47] and *R. percellens* [35]; the rays, *Gymnura poecilura* [39] and *Pateobatis bleekeri* (formerly *Dasyatis bleekeri*) [17]; the skate, *Sympterygia acuta* [48]; and the sharks, *Iago omanensis* [49], *Mustelus griseus*, *Mustelus manazo* [50] and *P. glauca* [51]; (2) a stratified ciliated cuboidal epithelium in the dogfish, *Squalus acanthias* [52]; and (3) a pseudostratified ciliated columnar epithelium in the shark, *Cetorhinus maximus* [19]; the rays, *Hypanus guttatus* (formerly *Dasyatis guttata*), *Narcine bancroftii*, *Urotrygon venezuelae* [35], *Hypanus sabinus* (formerly *Dasyatis sabina*) [45], *P. magdalenae* [42] and *F. signifer* in the present study. Further, the lining structure of the *F. signifer* ostium is a pseudostratified ciliated epithelium, unlike other chondrichthyans in which the ostium is bordered by simple or stratified ciliated columnar epithelia [1, 50]. It is noted that the oviductal epithelium of *F. signifer* produces and secretes neutral and acidic glycoproteins/mucopolysaccharides, similar to those in other chondrichthyans [17, 39, 42, 48, 49, 53]. These secretory materials may function in nurturing gametes and embryos and lubricating the oviductal lumen, thus possibly facilitating smooth passage of ovulated eggs towards the oviducal gland [52]. Additionally, the ciliary activity of the ciliated cells may be involved in transporting gametes and wafting secretory materials in the luminal milieu. Branching of the oviductal folds in *F. signifier*, similar to *C. maximus* [19], may increase surface areas of the oviductal mucosa for secretion and absorption.

Among chondrichthyans, the oviductal muscularis is constituted by different patterns of muscular placement, which vary interspecifically and can be categorized into six patterns: (1) the circular muscle scattered among collagenous tissues, as described in *P. magdalenae* [43], *Scyliorhinus canicula* (formerly *Scyllium canicula*) [54] and *F. signifer*; (2) the longitudinal muscle in *S. acuta* [48]; (3) both inner circular and outer longitudinal muscles, such as *I. omanensis* [49], *P. bleekeri* [17] and *S. acanthias* [52, 55]; (4) the inner circular, middle oblique and outer longitudinal muscle fibers, as in *H. colliei* [20]; (5) the irregularly-oriented muscle, as seen in *M. griseus* and *M. manazo* [50]; and (6) the absence of musculature, such as *G. poecilura* [39] and *R. lentiginosus* [47]. The outermost oviductal layer of *F. signifer* is the adventitia similar to other chondrichthyans [17, 42], while the serosa is the outermost layer in *M. griseus*, *M. manazo* [50] and *S. acanthias* [52]. High vascularization of the oviduct may be related to increased demands of the organ for consumption of oxygen and nutrients to drive metabolic activities of the oviductal cells, such as muscular contraction, ciliary beating and exocytosis of secretory materials from the epithelial cells.

The oviducal glands of *F. signifer* are not divisible into distinctive histological regions, similar to other myliobatiform elasmobranchs, such as *G. poecilura* [39], *P. magdalenae* [42] and *Urobatis jamaicensis* (formerly *Urolophus jamaicensis*) [38], while other chondrichthyans have four distinct histological zones of the oviducal glands, namely club, papillary, baffle and terminal zones [38, 56–58]. The secretory units of the *F. signifer* oviducal glands are formed mainly by simple unbranched tubular glands and to a lesser extent simple branched tubular glands, as in *Dipturus batis* (synonym of *Raja batis*) [59], but only simple unbranched tubular glands in *G. poecilura*, *L. erinacea*, *Mustelus canis*, *Raja eglanteria*, *S. canicula*, *Scyliorhinus stellaris*, *S. acanthias* and *U. jamaicensis* [37–39, 60, 61]. Consistently, the tubular gland units of the chondrichthyan oviducal glands are lined by a columnar epithelium composed of ciliated and secretory cells [38]. In an active secretory status, the secretory cell nuclei are disposed to the basal compartment likely due accumulation of cytoplasmic granules, thus displacing the nuclei to the eccentric position, similar to other chondrichthyans [38, 62]. Additionally, the muscularis displays interspecific variation in the muscular organization that can be classified into three patterns: (1) the sole circular musculature in *F. signifer*, similar to *Hemitriakis japonica* (formerly *Galeorhinus japonicus*) [63], *M. griseus*, *M. manazo* [50] and *P. magdalenae* [42]; (2) the only longitudinal muscle, as in *Etmopterus spinax* [64], *S. acuta* [48] and *S. bonapartii* [65]; and (3) no musculature in this layer, as seen in *M. canis*, *R. eglanteria*, *S. acanthias* and *U. jamaicensis* [60]. The presence of condensed nuclei in the secretory cells and the tubular gland lumina suggests that these cells undergo physiologic degeneration, rupture and subsequently discharge of their secretory granules and nuclei into the tubular lumina, probably indicating the mode of holocrine secretion besides the classical merocrine secretion of the secretory granules in the oviducal gland, as previously shown in *S. canicula* [66].

Non-uniformly spatial distribution of carbohydrates in the secretory granules within and among individual glandular tubules, as previously described [62], implies that the granules are not synchronously and homogeneously glycosylated, also pointing to their temporally dynamic production and secretion. Furthermore, differential glycoconjugate staining patterns of the granules among specimens may be related to reproductive statuses of specimens, as shown in *S. canicula* [67]. The oviducal glands of *F. signifer* do not contain glycogens and sulfated acid glycoproteins/mucopolysaccharides, unlike those of *S. canicula* showing zonal distribution of the two chemical groups [67]. Heterogeneous staining patterns of the granules upon AB pH 2.5 and pentachrome histochemistry are possibly due to altered stainability of the granules for the histologic dyes related to different levels of their glycosylation with carboxylated acid glycoconjugates.

The isthmus, which is a connecting segment between the oviducal gland and the uterus, has been shown in several chondrichthyan species [8, 10, 12, 18, 19, 68–72], but it is not found in *F. signifer*, similar to *G. poecilura* [39]. Three types of the uterine epithelia in adults have been identified with interspecific variability: (1) a pseudostratified columnar epithelium, as seen in *S. acuta* and *S. bonapartii* [14] and *F. signifer*; (2) a simple columnar epithelium in *H. colliei* [20], *L. erinacea*, *Rhizoprionodon terraenovae* [73] and *Sphyrna tiburo* [74]; and (3) a mixture of simple squamous and simple cuboidal epithelia in *Carcharhinus plumbeus* [75]. An extensively ramified system of subepithelial capillaries and thinning of the superficial uterine epithelial cells are similar to those in the uterine mucosa of *S. acanthias* during gestation [76]. These structures and their corresponding basement membrane have been proposed to establish “a pseudoplacental barrier” that serves as an interface and allows transport of diffusible materials between intraluminal and intramural compartments [76].

The presence of uterine trophonemata in *F. signifer* is typical of the suborder Myliobatoidei [15, 28, 32, 77]. Modification of the trophonematal microanatomy of *F. signifer* is likely related to their reproductive statuses and, in gestational females of other species, the trophonemata display fetal-maternal respiratory and trophic relationships [44]. In the non-gestational stage, the epithelium forms a continuous homeomorphic vestment along the entire trophonematal mucosa, whereas during the gestational stage the epithelium undergoes invagination to become glandular crypts, which are separated from one another by subepithelial vascular beds, as previously described [44, 78]. It has been documented that the trophonemata of the females bearing newly fertilized eggs are bordered by a simple cuboidal epithelium [44, 77]. Numerous vascularized trophonemata increase the surface areas for respiratory exchange, while intimate association between the attenuated epithelium and subepithelial capillaries minimizes diffusion distances between the trophonematal and intraluminal compartments, thereby effectively facilitating gas exchange between the female and the intrauterine young [15, 44, 77]. The glandular crypts secrete histotroph or uterine milk to nourish the intrauterine pups [15, 33, 43, 44]. This secretory material is of a serous type, as in other stingrays [2]. In addition, the uterine fluid may play a role in mammal-like sperm capacitation by increasing sperm motility, as described in the stingray, *P. motoro* [79]. Histological changes in the trophonematal epithelium of *F. signifer* are similar to those in *P. violacea* [80].

The myometrium of the chondrichthyan uterus consists of combinations of the muscular organization that have been classified into six patterns: (1) the inner circular and outer longitudinal muscle fibers, as in the rays, *G. poecilura* [39], *P. aiereba*, *P. iwamae*, *P. motoro*, *P.orbignyi*, *P. schroederi*, *P. scobina*, *P. wallacei* [41], *P. bleekeri* [6, 78], *P. magdalenae* [42], and *F. signifier*; the sharks, *M. griseus*, *M. manazo* [50] *M. schmitti* [69, 81] and *Scoliodon laticaudus* (formerly *S. sorrakowah*) [9]; the skates, *S. acuta* and *Sympterygia bonapartii* [14]; the ratfish, *H. colliei* [20] and the guitar fish, *R. lentiginosus* [47]; (2) the inner longitudinal and outer circular muscles, as described in the rays, *A. nichofii* [78], *B. walga* [82] and *L. erinacea* [73]; (3) three muscular tunics with a diagonal muscle layer as the outermost layer in the shark, *I. omanensis* [70]; (4) three muscular layers with the circular musculature intercalated between two layers of the longitudinal musculatures in *R. terraenovae* [83]; (5) three muscular layers, with the longitudinal muscle layer inserted between the inner and outer circular muscle layers, as in *S. tiburo* [74] and the topeshark, *H. japonica* [63]; and (6) uniquely in *Mustelus schmitti* having three muscular tunics: inner reticularly-orientated muscle, middle and outer longitudinal muscles [69]. Interstitial spaces in the myometrium of some specimens may accommodate imbibed water that is correlated with reproductive events, as proposed in the southern eagle ray, *Myliobatis goodei* [40].

Histological variation of the vaginal mucosa of *F. signifer* is attributed to disparate cell organization and histochemical properties of cytoplasmic granules. Morphological transformation of transitional-like superficial cells is likely due to accumulation and secretion statuses, while transitional epithelial cells of the mammalian urinary system undergo cell transformation in response to the filling status of the excretory passage [84]. Negative PAS reactivity of diastase-pretreated vaginal tissues of *F. signifer* indicates no glycogen production, unlike mammalian vaginal epithelial cells that synthesize and store the glycogens [85, 86]. Moreover, metachromatic property of the vaginal secretory granules upon toluidine blue staining is reported for the first time in the Chondrichthyes. Chemical structure(s) and role(s) of these granules remain unknown, and further study is required to unfold their structural and functional properties, possibly adding other functions of the vagina besides simply serving as a conduit for passage of gametes and full term embryos. The corrugated surface of the vagina due to mucosal folds may provide anchoring surfaces for the male claspers during copulation, as suggested previously [17].

Although the females collected at different months exhibit histological and histochemical variation of the genital ducts, it remains uncertain whether this variation is relevant to the annual reproductive cycle because the small sample size was collected each time and sampling did not occur throughout the full annual cycle. Regardless of body weights, body sizes and the presence of vitellogenic or postovulatory follicles, the lighter ovaries of F1, F6 and F7 females than those of the F2, F3, F4, F5 and F8 females likely reflect their less ovarian activity, i.e., folliculogenesis and steroidogenesis. Further, it has been suggested that ovarian steroids, like estradiol and progesterone, promote genital duct functions and morphological changes [87–89]. This may account for less complexity of tissue organization, histochemical profiles and secretory activities of the genital ducts in F1, F6 and F7 females, as compared to those of the other females.

## Conclusion

This article describes the microanatomy of female genital ducts of the freshwater dasyatid *Fluvitrygon signifer*, highlighting common and specific histological and histochemical characteristics. The anterior oviducts of mature specimens are actively secretory and have tall, branched mucosal folds, while those of immature/regenerating females form short, unbranched mucosal folds and show inactive secretion. The oviducal glands of *F. signifer* are inseparable into four histological zones, a typical feature of the Myliobatiformes. Immature/regenerating females have thin, inactive oviducal glands whereas the glands of mature females are constituted by actively secretory glandular tubules that are subdivided into three tubular patterns, based on chemical nature of secretory granules. The microanatomical structures of the uteri vary among specimens in terms of degrees of trophonematal and uterine vascularization, cell organization of trophonematal and uterine epithelia, and thickness of the uterine wall. The vaginal mucosa of immature/regenerating females contains fewer secretory granules, while those of mature specimens are loaded with the variously-sized granules, bearing different histochemical properties. Herein, comparative viewpoints on the microanatomy of the genital ducts among the Chondrichthyes have been made, thus contributing to better information on an evolutionary perspective of the chondricthyan reproduction. Moreover, the decline of *F. signifer* calls for development of protection plans for their wild populations. Basic knowledge on microanatomical structures of the female reproductive system of *F. signifer* in concert with other parameters, such as age, growth, reproductive cycle, reproductive endocrinology and reproductive behaviors, would be useful for formulating effective conservation strategies for this species.

## Methods

Eight females of *Fluvitrygon signifer* were collected from the Chao Phraya river, Nakhon Sawan province, central Thailand in 1999 and 2000 (Table 1). The specimens were identified based on Compagno and Roberts (1982) [28]. All live animals were immediately transported to the laboratory. Their body weights were determined using a balance to the nearest 1 g. Disc lengths, disc widths and tail lengths were measured using a measuring tape to the nearest 0.1 cm. They were anesthetized using 6.5 mM tricaine methanesulfonate as described by Campbell and Davies (1963) [90]. The thoracic cavity was cut open to expose the heart. They were sacrificed via transcardial perfusion using Bouin’s fixative through a polyethylene tube inserted into the conus arteriosus. The genital ducts (oviduct, oviducal gland, uterus and vagina) were collected and preserved in the same fixative for 24 h. They were dehydrated in a graded ethanol series, cleared in xylene, infiltrated and embedded in Paraplast Plus®, cut into 5 μm thick slices using a rotary microtome and stained with different histologic dyes: hematoxylin-eosin (H&E) for general histology, modified Movat’s pentachrome for differentiation among connective tissues, muscles and carboxylated acid glycoproteins/mucopolysaccharides, bromophenol blue for general proteins and toluidine blue for determining metachromasia of subcellular structures, alcian blue (AB) pH 1.0 and 2.5 for sulfated acid and carboxylated acid glycoproteins/mucopolysaccharides, respectively, periodic acid-Schiff (PAS) for neutral glycoproteins/mucopolysaccharides and hematoxylin for nuclear counterstaining (PAS-H), and PAS after pretreatment of tissue sections with 0.1% diastase (PAS-D) for differentiating glycogen from other PAS positive elements [91]. Histological slides were observed under a light microscope (Olympus BX51) and the photomicrographs were taken using a digital camera (Olympus DP70 Camera System). Sexual maturity was determined based on a combination of body morphometrics and ovarian histological examination, as previously described by Last et al. (2010), Last et al. (2016) [30, 31] and Somsap et al. (2019), Follesa et al., (2019) [34, 46], respectively (Table 1).

## Data Availability

The materials of *Fluvitrygon signifer* (embedded blocks, paraffin sections and stained histological slides) are stored at Department of Zoology, Faculty of Science, Kasetsart University. Raw data collected and all images taken are available from the corresponding author on reasonable request.

## Abbreviations

AB: Alcian blue
Ad: Adventitia
BC: Basal cell
CC: Ciliated cell
CD: Central duct
Ci: Cilia
CM: Circular muscle
CN: Condensed nucleus
Co: Columnar cell
CP: Connective tissue papilla
Cr: Glandular crypt
CT: Connective tissue
DC: Desquamated cell
Ep: Epithelium
Ga: Ganglion cell
Gr: Granule
GT: Glandular tubule
H&E: Hematoxylin and eosin
Lc: Leukocyte
LM: Longitudinal muscle
LP: Lamina propria
MC: Mucous cell
MF: Mucosal fold
Mu: Mucosa
Mus: Muscularis
OS: Ovarian surface
PAS: Periodic acid-Schiff
PAS-H: Periodic acid-Schiff-hematoxylin
PS: Peritoneal surface
SC: Secretory cell
Se: Secretory materials
SM: Smooth muscle
Tm: Trophonemata
UE: Uterine epithelium
Ut: Uterus
Va: Vagina
VE: Vaginal epithelium
*: Blood vessel
